# The value of myocardial work in the estimation of left ventricular systolic function in patients with coronary microvascular disease: A study based on adenosine stress echocardiography

**DOI:** 10.3389/fcvm.2023.1119785

**Published:** 2023-04-11

**Authors:** Quande Liu, Qimou Li, Xiaoyu Wan, Mingjun Xu, Jichen Pan, Yu Zhang, Mengmeng Li, Mei Zhang

**Affiliations:** The Key Laboratory of Cardiovascular Remodeling and Function Research, Chinese Ministry of Education, Chinese National Health Commission and Chinese Academy of Medical Sciences, the State and Shandong Province Joint Key Laboratory of Translational Cardiovascular Medicine, Department of Cardiology, Qilu Hospital, Cheeloo College of Medicine, Shandong University, Shandong, China

**Keywords:** myocardial work, adenosine stress echocardiography, coronary microvascular dysfunction, speckle tracking echocardiography, coronary flow reserve

## Abstract

**Background:**

Coronary microvascular dysfunction (CMD) is associated with increased cardiovascular events in patients with angina with non-obstructive coronary (ANOCA), especially heart failure. Conventional echocardiography is difficult to identify early alterations in cardiac function due to CMD.

**Methods:**

We recruited 78 ANOCA patients. All patients underwent conventional echocardiography examination, adenosine stress echocardiography and examination of coronary flow reserve (CFR) by transthoracic echocardiography. Based on the CFR results, patients were divided into the CMD group (CFR < 2.5) and the non-CMD group (CFVR ≥ 2.5). Demographic data, conventional echocardiographic parameters, two-dimensional speckle-tracking echocardiography (2D-STE) parameters and myocardial work (MW) were compared between the two groups at rest and at stress. Logistic regression was used to analyze the factors associated with CMD.

**Results:**

There was no significant difference in conventional echocardiography parameters, 2D-STE related indices or MW at rest between the two groups. Global work index (GWI), global contractive work (GCW), and global work efficiency (GWE) were lower in the CMD group than in the non-CMD group at stress (*p* = 0.040, 0.044, <0.001, respectively), but global waste work (GWW) and peak strain dispersion (PSD) were higher (both *p* < 0.001). GWI and GCW were associated with systolic blood pressure, diastolic blood pressure, product of heart rate and blood pressure, GLS and coronary flow velocity. While GWW was mainly correlated with PSD, GWE was correlated with PSD and GLS. In the non-CMD group, the responses to adenosine was mainly manifested as an increase in GWI, GCW and GWE (*p* = 0.001, 0.001, 0.009, respectively) and a decrease in PSD and GWW (*p* = 0.001, 0.015, respectively). In the CMD group, the response to adenosine was mainly manifested as an increase in GWW and a decrease in GWE (*p* = 0.002, and 0.006, respectively). In the multivariate regression analysis, we found that ΔGWW (difference in GWW before vs. after adenosine stress) and ΔPSD (difference in PSD before vs. after adenosine stress) were independent factors associated with CMD. The ROC curves showed that the composite prediction model consisting of ΔGWW and ΔPSD had excellent diagnostic value for CMD (area under the curve = 0.913).

**Conclusion:**

In the present study, we found that CMD caused deterioration of myocardial work in ANOCA patients under adenosine stress, and that increased cardiac contraction asynchrony and wasted work may be the main changes caused by CMD.

## 1. Introduction

Approximately 112 million people worldwide suffer from angina, but nearly half of the patients who undergo coronary angiography for angina do not have significant coronary stenosis, they are referred to as angina with non-obstructive coronary arteries (ANOCA) (1). Previous studies have confirmed that patients with ANOCA have a higher risk of cardiovascular events than asymptomatic healthy populations (2), therefore, ANOCA has been getting more attention. Recent investigations indicated that up to two-thirds of patients with ANOCA had coronary microvascular dysfunction (CMD) (3). CMD is reported to be strongly associated with an increased risk of major adverse cardiovascular events. A meta-analysis involving 6,631 patients with ANOCA showed that patients with CMD had higher rates of mortality and major cardiovascular events than those without CMD, and heart failure is considered the major adverse cardiovascular event caused by CMD (4). Therefore, the detection of CMD and the early recognition of its effects on cardiac systolic function are of vital importance.

The diagnosis of CMD usually relies on the functional assessment of microcirculation, which includes invasive and noninvasive methods. The measurement of coronary flow reserve (CFR) using transthoracic echocardiography is one of the most widely used methods because of its convenience and reliability in the clinic. In addition, echocardiography can simultaneously assess cardiac systolic function. Previous studies have confirmed that CMD is associated with elevated markers of myocardial injury and myocardial ischemia (5), which are also thought to contribute to poor prognosis. Even though patients with CMD have significant abnormalities, conventional echocardiography does not identify early changes in cardiac systolic function and stress echocardiography rarely demonstrates regional wall motion abnormalities. Therefore, more sensitive indicators for early detection of cardiac dysfunction in patients with CMD are urgently needed.

Two-dimensional (2D) speckle-tracking echocardiography (STE) myocardial global longitudinal strain (GLS) can detect subclinical changes in cardiac systolic function when left ventricular ejection fraction (LVEF) is normal and is better than LVEF at predicting cardiovascular events (6). 2D-STE peak strain dispersion (PSD), which refers to the standard deviation of the peak time of longitudinal strain in each LV segment, accurately reflects the coordination of cardiac motion and is often used to assess LV synchrony (7).

However, 2D-STE GLS is load dependent, which limits its use in certain hemodynamic conditions, such as hypertension. Current speckle-tracking techniques can calculate the myocardial work index (MW) by integrating longitudinal strain and arterial blood pressure to obtain a noninvasive left ventricular pressure-strain loop (LV-PSL) (8). Noninvasive assessment of MW is a novel modality that has been investigated in several studies of cardiac conditions, such as judging patient response to cardiac resynchronization therapy and predicting the prognosis of ST-segment elevation myocardial infarction (9, 10). MW has also demonstrated exceptional value in identifying subclinical changes in LV systolic function due to hypertension (11, 12). However, the application value of MW in CMD has been little explored.

Therefore, we investigated the effect of CMD on cardiac function represented by MW in ANOCA patients, and explored the predictive ability of LV mechanicals parameters on CMD.

## 2. Methods

### 2.1. Patients

We recruited 78 ANOCA patients referred for adenosine-based transthoracic Doppler echocardiography-assessed coronary flow reserve (CFR) measurement, adenosine stress echocardiography and conventional echocardiography. Diagnostic criteria of ANOCA: (1) the patient had symptoms of angina or angina equivalents; (2) coronary angiography or coronary CT angiography suggested coronary artery stenosis was <50% (13); (3) Objective evidence of myocardial ischemia. Exclusion criteria: (1) suboptimal image quality; (2) left ventricular ejection fraction (LVEF) <50%; (3) atrial fibrillation or other severe arrhythmias; (4) severe valvular disease; and (5) intracardiac shunt. All patients are requested to stop taking medications that may affect the test results the day before the test. According to the International Standardization of Diagnostic Criteria for Microvascular Angina issued by Coronary Vasomotion Disorders International Study Group, CMD was defined as CFR < 2.5 (13). The study was approved by the Ethics Committee of Scientific Research of Shandong University Qilu Hospital (KYLL202008019) and was conducted as per the Declaration of Helsinki. All patients were informed about the study.

### 2.2. 2D echocardiography, pulsed-wave Doppler and tissue Doppler imaging

A GE Vivid E95/E9 ultrasound diagnostic apparatus with an M5S probe (3.5 MHz) (GE Medical Systems, Milwaukee, WI, United States) was used. Brachial artery blood pressure was measured prior to image acquisition. All subjects were connected simultaneously to a thoracic-lead ECG. Parasternal left ventricular long-axis views, and apical four-chamber, three-chamber, and two-chamber views were acquired continuously for at least three cardiac cycles in the left lateral recumbent position at a frame rate of 50–80 frames/s. We measured conventional echocardiographic parameters, including: LV end-diastolic dimension (LVEDD), LV end-systolic dimension (LVESD), LV interventricular septal end-diastolic thickness (IVST), LV posterior wall thickness (LVPWT), LV mass index (LVMI), left atrium volume index (LAVI), rest LV end-diastolic volume index (rest-LVEDVI), rest LV end-systolic volume index (rest-LVESVI), and rest LV ejection fraction (rest-LVEF, Simpson's biplane method). Pulsed-wave Doppler (PW) and tissue Doppler imaging (TDI) of the mitral valve were also evaluated.

### 2.3. Adenosine stress echocardiography

Patients underwent adenosine stress echocardiography according to the protocol recommended by the EACVI (European Association of Cardiovascular Imaging) (14). We used adenosine at a dose of 0.14 mg/kg/min over 6 min. The electrocardiogram was monitored continuously and blood pressure was monitored intermittently. Criteria for interrupting the test were severe chest pain, diagnostic ST-segment shift, excessive blood pressure increase (systolic blood pressure ≥240 mmHg, diastolic blood pressure ≥120 mmHg), dyspnea, hypotension (systolic blood pressure ≤90 mmHg, diastolic blood pressure ≤60 mmHg), maximal predicted heart rate, or significant arrhythmias. When the adenosine stress was maximal, that is, when the coronary blood flow velocity was maximal, the three apical views (the apical four-chamber, two-chamber, and long axis) were repeatedly recorded and the cardiac systolic function related indices were measured, including peak-LVEDVI, peak-LVESVI and peak LVEF.

### 2.4. Coronary flow reserve

Coronary flow reserve testing was performed using a previously published and validated protocol (15). The mid-distal segment of the left anterior descending (LAD) branch was identified in the interventricular sulcus under a modified left ventricular double-chamber view, and the flow spectrum was recorded by color Doppler echocardiography to detect the mid-distal coronary artery flow in the left anterior descending branch. CFR was defined as the ratio of the maximum diastolic flow velocity in the hyperemic state to the maximum diastolic flow velocity in the basal state.

### 2.5. Speckle-tracking echocardiography

Speckle-tracking echocardiography was performed using an offline workstation (EchoPAC version 204; GE Vingmed Ultrasound AS, GE Medical Systems) to calculate 2D-STE parameters. Based on three apical views, the software automatically identifies myocardial activity in the region of interest to enable automatic tracking of myocardial motion. If necessary, the region of interest was adjusted by correcting the edge of the endocardium or the width of the myocardium. According to the standardized 17-segment heart model (16), GLS was calculated from the mean of the longitudinal peak systolic strain of all the LV segments. From the time to reach the peak strain in each segment, the software automatically calculated the PSD.

### 2.6. Myocardial work

To calculate MW-related indices, mitral and aortic valve opening and closing times were first determined in the apical three-chamber cardiac section, followed by inputting brachial artery systolic pressure to replace the peak LV pressure to obtain the noninvasive LV pressure-strain loop (LV-PSL). Based on LV-PSL, the following data were obtained: global work index (GWI), global contractive work (GCW), global waste work (GWW) and global work efficiency (GWE).

2D-STE and MW-related indices were measured before adenosine stress and at maximum adenosine stress, where pre-adenosine stress was defined as rest and post-adenosine stress was defined as peak. The difference before and after adenosine stress was calculated as Δ.

### 2.7. Intra- and interobserver variability

Two experienced sonographers remeasured 20 randomly selected participants to assess the repeatability. The sonographers were blinded to the clinical data as well as to each other's results. A month later, the images were analyzed again by the same sonographers to assess the intraobserver variability and to assess the interobserver variability.

### 2.8. Statistical analysis

All data were collected, statistically analyzed, and tabulated using SPSS 26 software (SPSS Inc., Chicago, IL, United States), Med Calc 19.04 (Med Calc Software BVBA, Ostend, Belgium) and GraphPad Prism 9.0.0 (GraphPad Software, San Diego, CA, United States). Baseline variable statistical significance was assessed with the Wilcoxon test, analysis of variance, and the *χ*^2^ test for continuous nonnormally distributed, continuous normal distributed, and categorical variables, respectively. Normally distributed continuous variables are presented as mean ± SD. Nonnormally distributed continuous variables are presented as median [*Q*1, *Q*3]. Categorical variables are presented as number (%). Pearson's correlation method was used to explore the variables influencing MW indices. Univariable and multivariable logistic regression analyses were performed to find the variables associated with CMD. The receiver operating characteristic (ROC) curve was drawn to analyze the predictive ability of 2D-STE and MW indices for CMD and to calculate the area under the curve (AUC). A value of *p* < 0.05 was considered significant.

## 3. Results

### 3.1. Study population and clinical characteristics

**Figure 1:**
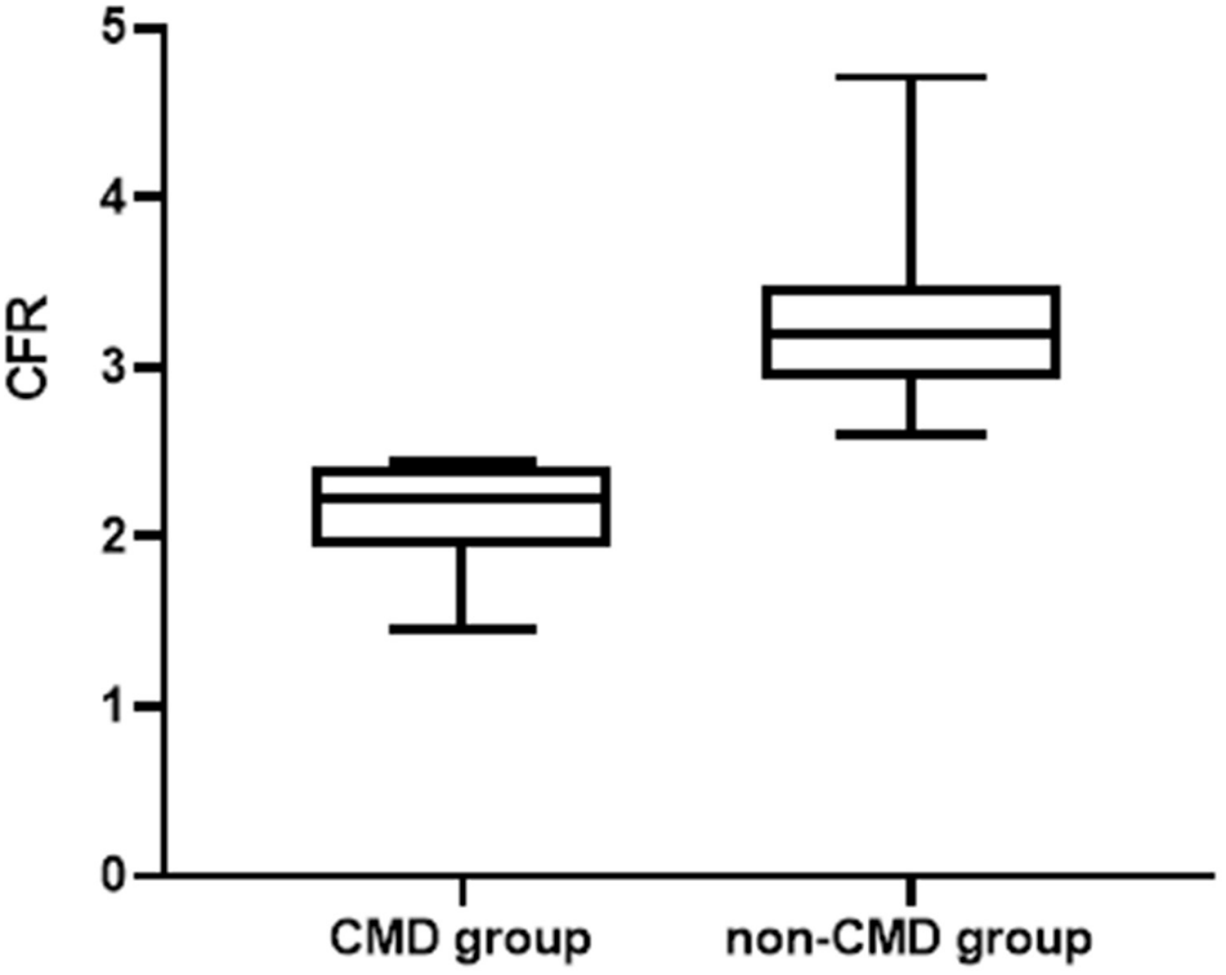
Comparison of CFR between the two groups. CFR, coronary flow reserve; CMD, coronary microvascular dysfunction.

The study population comprised 78 subjects with ANOCA. The average age of the entire population was 54.3 ± 10.3 years old; 28% of the subjects were female. The prevalence of CFR < 2.5, consistent with CMD, was 41% (32/78 subjects), and 59% (46/78 subjects) had CFR ≥ 2.5, consistent with non-CMD (Figure 1). Baseline demographics, cardiac risk factors, and relevant medications are compared by the presence of CMD in Table 1. The level of HDL-C was significantly higher in the non-CMD group than in the CMD groups. All other baseline clinical variables were not significantly different between the two groups.

**Table 1:** Clinical characteristics of the patients with or without CMD.

Clinical characteristics	Non-CMD group (*N* = 46)	CMD group (*N* = 32)	*p*-value
Age (years)	53.8 ± 9.8	55.1 ± 11.0	0.592
Female, *n* (%)	12 (26.1)	10 (31.3)	0.618
Comorbidities, *n* (%)			
Hypertension	20 (43.5)	16 (50)	0.570
Hyperlipidemia	26 (56.5)	24 (75)	0.094
Diabetes	9 (19.6)	8 (25)	0.567
Obesity	8 (17.4)	5 (15.6)	0.837
Alcohol drinker	21 (45.7)	17 (48.6)	0.516
Cigarette smoker@Medications, *n* (%)	24 (52.2)	16 (50)	0.850
Aspirin	34 (73.9)	28 (87.5)	0.144
β-blocker	15 (32.6)	19 (59.4)	0.019
Calcium-channel blocker	14 (30.4)	6 (18.8)	0.245
Statin	33 (71.7)	26 (81.3)	0.336
ACE-inhibitor or ARB	18 (3.1)	14 (43.8)	0.683
Trimetazidine	9 (19.6)	9 (28.1)	0.377
Nitrates	12 (26.1)	9 (28.1)	0.842
Nicorandil	10 (21.7)	16 (50)	0.009
Laboratory data			
Cholesterol (mmol/L)	4.1 (3.3, 4.7)	4.0 (3.1, 4.4)	0.490
HDL-C (mmol/L)	1.3 (1.1, 1.5)	1.2 (0.9, 1.4)	0.042
LDL-C (mmol/L)	2.2 (1.6, 3.0)	2.3 (1.7, 2.7)	0.951
BUN (mmol/L)	5.4 ± 1.3	5.3 ± 1.3	0.618
Creatinine (µmol/L)	71 (65, 76)	69.3 (63, 76)	0.355
Creatine kinase (IU/L)	90.8 (72, 96)	83.5 (66.5, 91.7)	0.316
Vital signs and physical characteristics			
Body mass index (kg/m^2^)	25.1 ± 3.5	25.4 ± 3.6	0.815
Body surface area (m^2^)	1.8 ± 0.2	1.8 ± 0.2	0.582
HRR	1.2 ± 0.2	1.2 ± 0.3	0.640
Abnormal HRR, *n* (%)	26 (56.5)	21 (65.6)	0.419
Heart structure			
LVEDD (mm)	45.2 ± 5.3	46.0 ± 6.3	0.544
LVESD (mm)	31.4 ± 5.3	33.9 ± 5.1	0.051
IVST (mm)	10.7 ± 2.5	10.1 ± 1.9	0.246
LVPWT (mm)	10.2 ± 2.1	9.4 ± 1.6	0.079
LVMI (g/m^2^)	91.4 ± 20.5	87.5 ± 23.5	0.436
LAVI (ml/m^2^)	19.1 ± 6.2	20.3 ± 7.5	0.420
Pulsed-wave Doppler indices			
E velocity (m/s)	0.7 ± 0.1	0.7 ± 0.2	0.729
A velocity (m/s)	0.7 ± 0.2	0.8 ± 0.2	0.076
E/A ratio	1.0 ± 0.3	1.0 ± 0.3	0.116
Tissue Doppler indices			
Septal *s*′ velocity (m/s)	0.07 ± 0.02	0.07 ± 0.02	0.867
Septal *e*′ velocity (m/s)	0.07 ± 0.02	0.06 ± 0.02	0.057
Septal *a*′ velocity (m/s)	0.09 ± 0.02	0.09 ± 0.02	0.678
E/e′	8.3 ± 2.1	9.6 ± 2.7	0.025
Coronary blood flow velocity (m/s)			
Rest flow velocity	0.20 ± 0.05	0.23 ± 0.08	0.023
Peak flow velocity	0.64 ± 0.17	0.49 ± 0.18	0.001

CMD, coronary microvascular dysfunction; ACE, angiotensin converting enzyme; ARB, angiotensin receptor blocker; HRR, heart rate reserve; HDL-C, high density lipoprotein cholesterol; LDL-C, low density lipoprotein cholesterol; BUN, blood urea nitrogen; LVEDD, left ventricular end-diastolic dimension; LVESD, left ventricular end-systolic dimension; IVST, interventricular septal end-diastolic thickness; LVPWT, left ventricular posterior wall thickness; LVMI, left ventricular mass index; LAVI, left atrium volume index.

### 3.2. Echocardiographic characteristics

Parameters by 2D echocardiography, PW, and TDI, including LVEDD, LVESD, E velocity and coronary flow velocity were compared between the CMD group and non-CMD group (Table 1). Except for the higher *E*/*e*′ in the CMD group, there were no significant differences in the 2D, PW or TDI indicators between the two groups. In addition, the resting flow velocity of the CMD group was faster, and the peak coronary flow velocity was slower.

After adenosine stress, the absolute values of GLS and LVEF were significantly increased in all groups. In the CMD group, GWW increased significantly and GWE decreased significantly, but no significant changes were observed in the remaining indicators. In the non-CMD group, there was a significant decrease in PSD and GWW along with a significant increase in GWI, GCW and GWE. Regarding hemodynamic parameters, heart rate increased significantly after adenosine stress in both groups. While, SBP and DBP decreased significantly after adenosine stress in the non-CMD group, but not in the CMD group (Table 2).

**Table 2:** Comparison of cardiac contractility between the two groups.

Parameters	Non-CMD group (*N* = 46)	CMD group (*N* = 32)	*p*-value
**Systolic blood pressure (mmHg)**			
Rest	130.2 ± 14.3	131.8 ± 17.2	0.655
Peak	119.9 ± 15.3*	127.3 ± 22.9	0.092
ΔSystolic blood pressure (mmHg)	−10.2 ± 12.7	−4.5 ± 16.5	0.085
**Diastolic blood pressure (mmHg)**			
Rest	81.5 ± 10.3	81 ± 14.8	0.867
Peak	74.6 ± 11.0*	76.9 ± 14.8	0.432
ΔDiastolic blood pressure (mmHg)	−6.9 ± 9.7	−4.1 ± 10.7	0.234
**Heart rate (bpm)**			
Rest	69.8 ± 10.1	70.9 ± 12.1	0.657
Peak	87.9 ± 11.9*	86.5 ± 13.4*	0.638
ΔHeart rate (bpm)	18.1 ± 11.9	15.6 ± 11.4	0.360
**RPP**			
Rest	8,992 (7,502, 10,138)	9,246 (8,117, 10,224)	0.696
Peak	10,391 (8,800, 11,700)	10,230.5 (9,407, 11,655)	0.622
ΔRPP	1,485.1 ± 1,828.7	1,708.5 ± 2,031.6	0.614
**LVEDVI (ml/m^2^)**			
Rest	39.4 ± 7.0	37.2 ± 9.1	0.221
Peak	38.9 ± 7.4	39.0 ± 7.6	0.966
ΔLVEDVI (ml/m^2^)	−0.5 ± 5.6	1.8 ± 5.2	0.067
**LVESVI (ml/m^2^)**			
Rest	12.9 ± 3.9	12.4 ± 3.7	0.628
Peak	10.5 ± 3.6*	10.8 ± 3.5	0.714
ΔLVESVI (ml/m^2^)	−2.3 ± 3.3	−1.6 ± 2.8	0.314
**LVEF (%)**			
Rest	67.5 ± 6.3	66.3 ± 5.5	0.411
Peak	73.1 ± 6.0*	72.8 ± 6.3*	0.846
ΔLVEF (%)	5.6 ± 7.2	6.5 ± 5.5	0.566
**GLS (%)**			
Rest	−21.7 ± 2.9	−20.6 ± 2.6	0.086
Peak	−26.9 ± 4.5*	−23.0 ± 3.9*	0.001
ΔGLS (%)	−5.2 ± 3.8	−2.4 ± 3.0	<0.001
**PSD (ms)**			
Rest	37.6 ± 13.6	38.8 ± 13.4	0.7115
Peak	28.5 ± 12.9*	45.4 ± 16.4	<0.001
ΔPSD (ms)	−9.2 ± 10.5	6.7 ± 10.8	<0.001
**Global work index (mmHg%)**			
Rest	2,182.5 ± 419.3	2,087.5 ± 478.3	0.356
Peak	2,502.1 ± 460.6*	2,252.4 ± 608.9	0.040
ΔGlobal work index (mmHg%)	322.7 ± 384.5	164.9 ± 432.3	0.094
**Global contractive work (mmHg%)**			
Rest	2,529.3 ± 473.3	2,413.2 ± 521.6	0.310
Peak	2,877.5 ± 549.6*	2,600.9 ± 634.3	0.044
ΔGlobal contractive work (mmHg%)	348.2 ± 457.9	187.7 ± 441.9	0.127
**Global waste work (mmHg%)**			
Rest	76.3 ± 52.4	77.2 ± 79.6	0.951
Peak	39 (24, 86)*	103.5 (72, 185)*	<0.001
ΔGlobal waste work (mmHg%)	−11(−36, 17)	42 (13.5, 138.5)	<0.001
**Global work efficiency (mmHg%)**			
Rest	97 (96, 98)	97 (95.5, 98)	0.432
Peak	98 (96, 95)*	95 (92, 97)*	<0.001
ΔGlobal work efficiency (mmHg%)	1 (0, 2)	−2(−3.5, 0)	<0.001

RPP, product of heart rate and blood pressure; LVEDVI, left ventricular end-diastolic volume index; LVESVI, left ventricular end-systolic volume index; LVEF, left ventricular ejection fraction; GLS, global longitudinal strain; PSD, peak strain dispersion.

* *p* < 0.05 VS rest indices.

**Figure 2:**
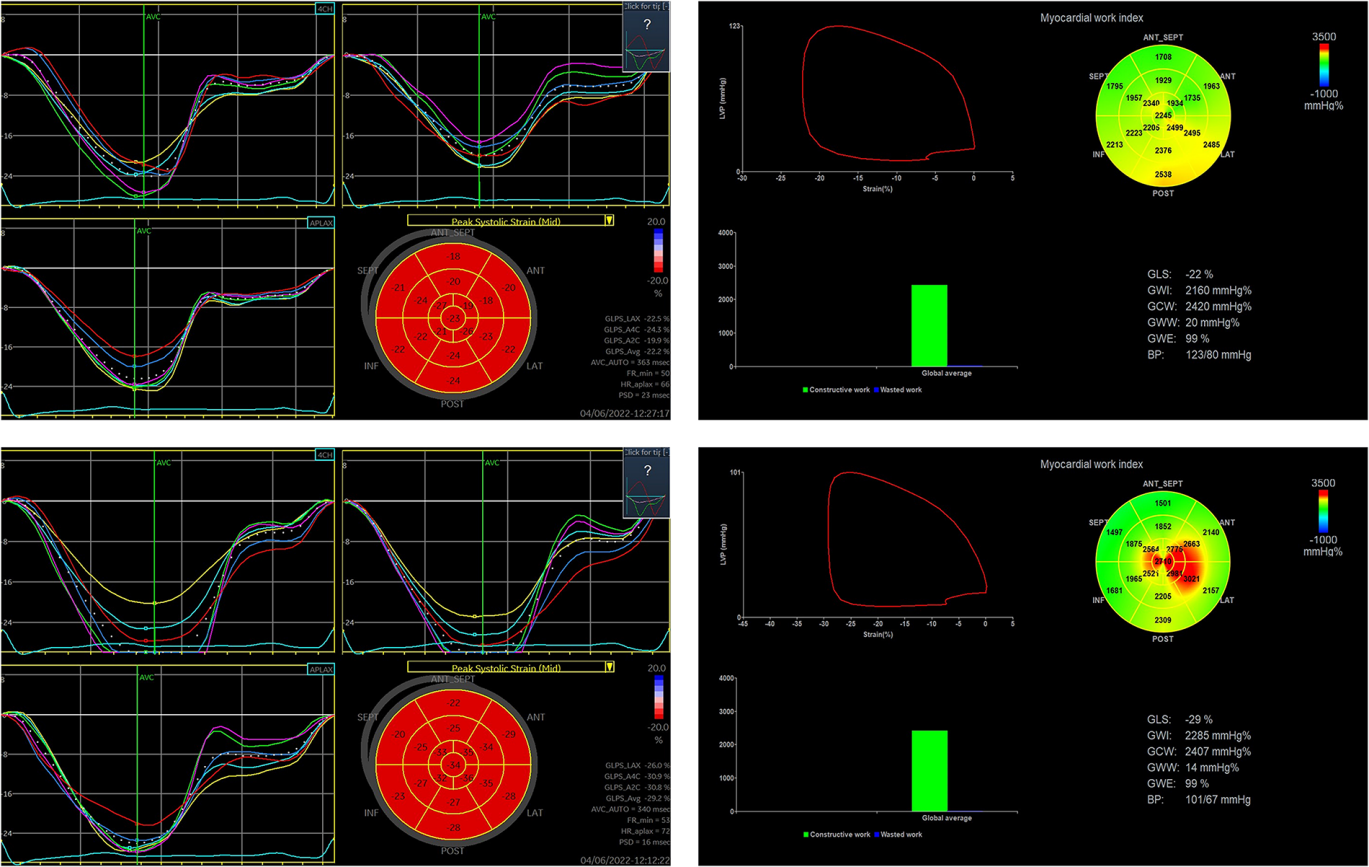
Changes in 2D-STE-related indexes and MW before and after adenosine stress in a patient in the non-CMD group. (The upper row shows 2D-STE related indices and MW at rest and the lower row shows 2D-STE related indices and MW at stress in ANOCA patient without CMD.) (This patient was a 57-year-old woman, 1.68 m tall, 65 kg, with a blood pressure of 123/80 mmHg before and 101/67 mmHg after adenosine stress, who was treated with aspirin, statin and metoprolol before examination.).

**Figure 3:**
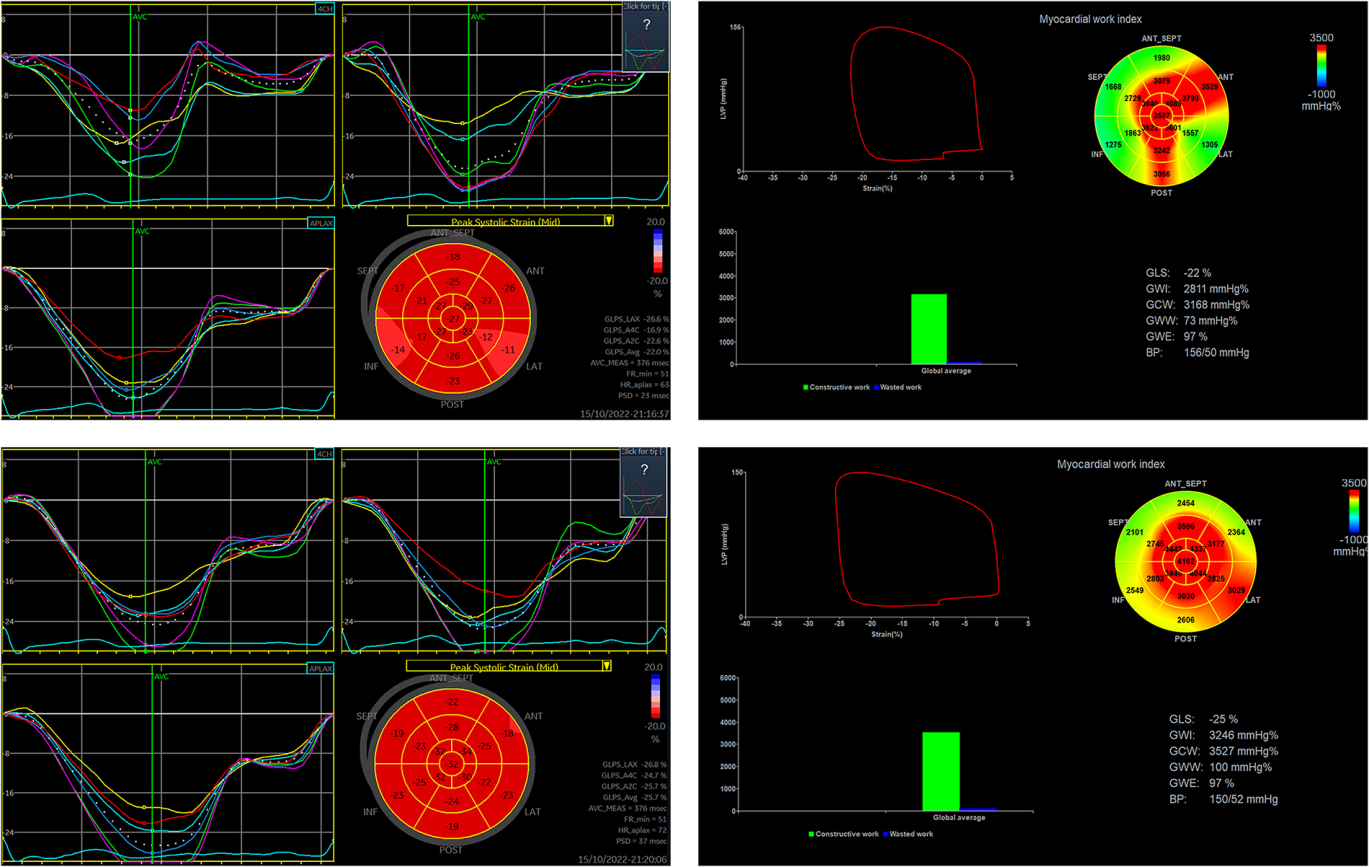
Changes in 2D-STE-related indexes and MW before and after adenosine stress in a patient in the CMD group. (The upper row shows 2D-STE related indices and MW at rest and the lower row shows 2D-STE related indices and MW at stress in ANOCA patient with CMD) (This patient was a 59-year-old woman, 1.66 m tall, 71 kg, with a blood pressure of 127/76 mmHg before and 102/64 mmHg after adenosine stress, who was treated with aspirin, statin and nicorandil before examination).

In the comparison of cardiac contractility metrics between the two groups, the differences appeared mainly after adenosine stress. Of these, the absolute values of peak GLS and ΔGLS were significantly lower in the CMD group than in the non-CMD group. The peak PSD and ΔPSD in the CMD group were significantly higher than those in the non-CMD group. The CMD group had lower peak GWI, peak GCW, and peak GWE, but higher peak GWW and ΔGWW (Figures 2, 3 and Table 2).

### 3.3. Analyses of factors associated with MW indices

GWI was significantly correlated with SBP, DBP, the product of heart rate and blood pressure (RPP), GLS and coronary flow velocity at rest and under stress and significantly correlated with PSD, LVEF and LVESVI only under stress. The factors affecting GCW, both at rest and under stress, were the same as those affecting GWI, except that GCW at rest was correlated with LVEF. GWW was significantly associated with PSD in both the rest and stress states, while GWE was significantly associated with PSD and GLS in both the rest and stress states (Table 3).

**Table 3:** Correlation of hemodynamic, 2D-STE, and conventional echocardiographic parameters with MW-related parameters at rest and at stress.

Parameters	Global work index				Global contractive work				Global waste work				Global work efficiency			
	Rest		Peak		Rest		Peak		Rest		Peak		Rest		Peak	
	*r*	*p*	*r*	*p*	*r*	*p*	*r*	*p*	*r*	*p*	*r*	*p*	*r*	*p*	*r*	*p*
Systolic blood pressure	0.702	<0.001	0.514	<.001	0.667	<0.001	0.475	<0.001	0.028	0.809	−0.003	0.980	0.080	0.484	0.138	0.229
Diastolic blood pressure	0.419	<0.001	0.328	0.003	0.414	<0.001	0.360	0.001	0.068	0.553	0.095	0.408	0.015	0.897	0.022	0.852
Heart rate	−0.107	0.351	0.074	0.522	−0.087	0.448	0.089	0.438	0.178	0.119	0.074	0.521	−0.180	0.114	−0.071	0.538
RPP	0.340	0.002	0.397	<0.001	0.339	0.002	0.376	0.001	0.154	0.178	0.039	0.732	−0.089	0.437	0.048	0.674
GLS	−0.694	<0.001	−0.632	<0.001	−0.743	<0.001	−0.661	<0.001	0.158	0.166	0.186	0.104	−0.378	0.001	−0.444	<0.001
PSD	−0.098	0.392	−0.252	0.026	−0.163	0.154	−0.300	0.008	0.445	<0.001	0.423	<0.001	−0.569	<0.001	−0.596	<0.001
LVEF	0.205	0.072	0.298	0.008	0.227	0.046	0.330	0.003	0.087	0.450	0.157	0.170	−0.023	0.842	−0.022	0.848
LVEDVI	−0.010	0.932	−0.046	0.689	−0.003	0.976	−0.124	0.281	−0.245	0.030	−0.116	0.311	0.140	0.223	0.054	0.637
LVESVI	−0.143	0.213	−0.233	0.040	−0.149	0.193	−0.314	0.005	−0.169	0.139	−0.160	0.161	0.066	0.564	0.026	0.825
Coronary flow velocity	0.229	0.044	0.253	0.026	0.222	0.050	0.259	0.022	0.180	0.116	0.078	0.498	−0.049	0.669	0.044	0.699

SBP, systolic blood pressure; DBP, diastolic blood pressure; HR, heart rate; RPP, product of heart rate and blood pressure; GLS, global longitudinal strain; PSD, peak strain dispersion; LVEF, left ventricular ejection fraction; LVEDVI, left ventricular end-diastolic volume index; LVESVI, left ventricular end-systolic volume index.

Supplementary Table S1 shows the factors affecting ΔMW. ΔGWI was significantly correlated with ΔSBP, ΔDBP, ΔHR, ΔRPP and ΔGLS. GCW was significantly correlated with ΔSBP, ΔDBP, ΔHR, ΔGLS and ΔLVESVI. Both ΔGWW and ΔGWE were associated with ΔPSD, but ΔGWE was also associated with ΔSBP and ΔGLS.

Previous studies have shown that SBP is associated with GWI only in hypertensive patients, but in our study, SBP was associated with GWI not only in ANOCA patients with hypertension (rest: *r* = 0.695, *p* < 0.001, peak: *r* = 0.489, *p* = 0.002) but also in ANOCA patients without hypertension (rest: *r* = 0.727, *p* < 0.001, peak: *r* = 0.517, *p* < 0.001). And this correlation was also seen in ΔSBP and ΔGWI (hypertension group: *r* = 0.577, *p* < 0.001, non-hypertension group: *r* = 0.586, *p* < 0.001).

### 3.3. Predictors of CMD

The significant predictors of CMD on univariable regression analysis were *E*/*e*′, peak GLS, ΔGLS, peak PSD, ΔPSD, peak GWI, peak GCW, peak GWW, ΔGWW, peak GWE and ΔGWE. The significant predictors of CMD on multivariable regression analysis were ΔPSD and ΔGWW (Table 4). We also performed multivariable modeling that adjusted for age, sex, diabetes mellitus, hyperlipidemia, obesity, and hypertension. ΔPSD and ΔGWW remained significantly correlated with CMD (*p* = 0.024, OR = 1.165; *p* = 0.020, OR = 1.037, respectively) after controlling for these factors. To eliminate the effect of blood pressure, we performed multivariable modeling that adjusted for stress-SBP and stress-DBP. ΔPSD and ΔGWW remained significantly correlated with CMD (*p* = 0.043, OR = 1.124; *p* = 0.015, OR = 1.031, respectively).

**Figure 4:**
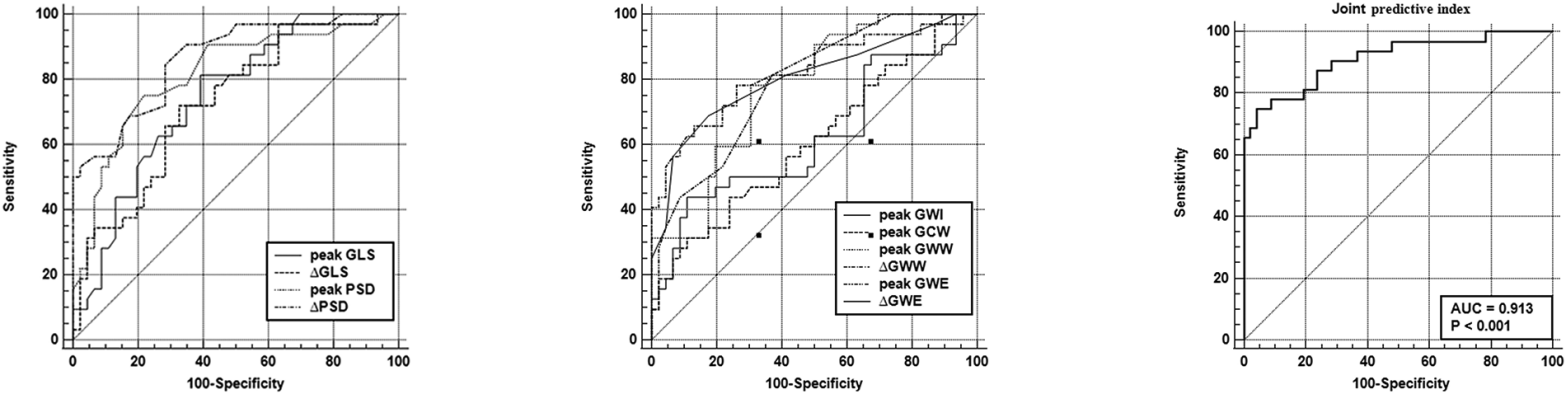
Receiver operating characteristic analysis of 2D-STE related indices, MW related indices and combined forecast metrics for predicting CMD. GLS, global longitudinal strain; PSD, peak strain dispersion; GWI, global work index; GCW, global contractive work; GWW, global waste work; GWE, global work efficiency.

According to ROC analysis, 2D-STE and MW indicators have diagnostic value for CMD, with GWW and PSD having the larger AUCs (Figure 4 and Table 5). To improve the diagnostic efficacy for CMD, we combined ΔPSD and ΔGWW into a new joint predictive index, which showed stronger predictive performance (AUC: 0.913, sensitivity: 75%, specificity: 95.65%).

**Table 4:** Parameters linked with CMD.

Parameters	Univariate Analysis		Multivariate Analysis	
	Odd Ratio (95% CI)	*p*	Odd Ratio (95% CI)	*p*
HDL-C	0.479 (0.180–1.274)	0.140		
E/e′	1.255 (1.022–1.540)	0.030	1.141 (0.688–1.893)	0.609
Peak GLS	1.251 (1.101–1.422)	0.001	1.361 (0.935–1.982)	0.108
ΔGLS	1.313 (1.109–1.555)	0.002	1.084 (0.738–1.593)	0.680
Peak PSD	1.093 (1.045–1.144)	<0.001	1.056 (0.965–1.155)	0.235
ΔPSD	1.181 (1.093–1.276)	<0.001	1.138 (1.024–1.265)	0.017
Peak Global work index	0.999 (0.998–1.000)	0.045	1.002 (0.999–1.004)	0.223
Peak Global contractive work	0.999 (0.998–1.000)	0.048		
Peak Global waste work	1.016 (1.007–1.026)	0.001	1.002 (0.983–1.022)	0.823
ΔGlobal waste work	1.025 (1.011–1.039)	<0.001	1.031 (1.007–1.055)	0.012
Peak Global work efficiency	0.678 (0.544–0.846)	0.001		
ΔGlobal work efficiency	0.542 (0.402–0.740)	<0.001		

HDL-C, high density lipoprotein cholesterol; GLS, global longitudinal strain; PSD, peak strain dispersion.

**Table 5:** ROC curve analysis for the detection of CMD.

Parameters	AUC (95% CI)	*p*-value	Cut-off point	Sensitivity	Specificity
Peak GLS	0.743 (0.632–0.835)	<0.001	>−26.6	81.2	60.9
ΔGLS	0.727 (0.614–0.821)	<0.001	>−3.7	71.9	61.4
Peak PSD	0.815 (0.710–0.894)	<0.001	>36	75	78.3
ΔPSD	0.863 (0.767–0.930)	<0.001	>−4	84.4	71.7
Peak Global work index	0.622 (0.505–0.730)	0.072	≤2,042	43.7	89.1
Peak Global contractive work	0.610 (0.493–0.719)	0.096	≤2,309	31.25	89.13
Peak Global waste work	0.776 (0.667–0.863)	<0.001	>61	78.12	69.57
ΔGlobal waste work	0.821 (0.717–0.898)	<0.001	>23	65.62	86.96
Peak Global work efficiency	0.787 (0.679–0.871)	<0.001	≤97	81.25	63.04
ΔGlobal work efficiency	0.803 (0.698–0.885)	<0.001	≤−1	68.75	82.61

GLS, global longitudinal strain; PSD, peak strain dispersion.

### 3.4. Intra- and interobserver reliability

**Figure 5:**
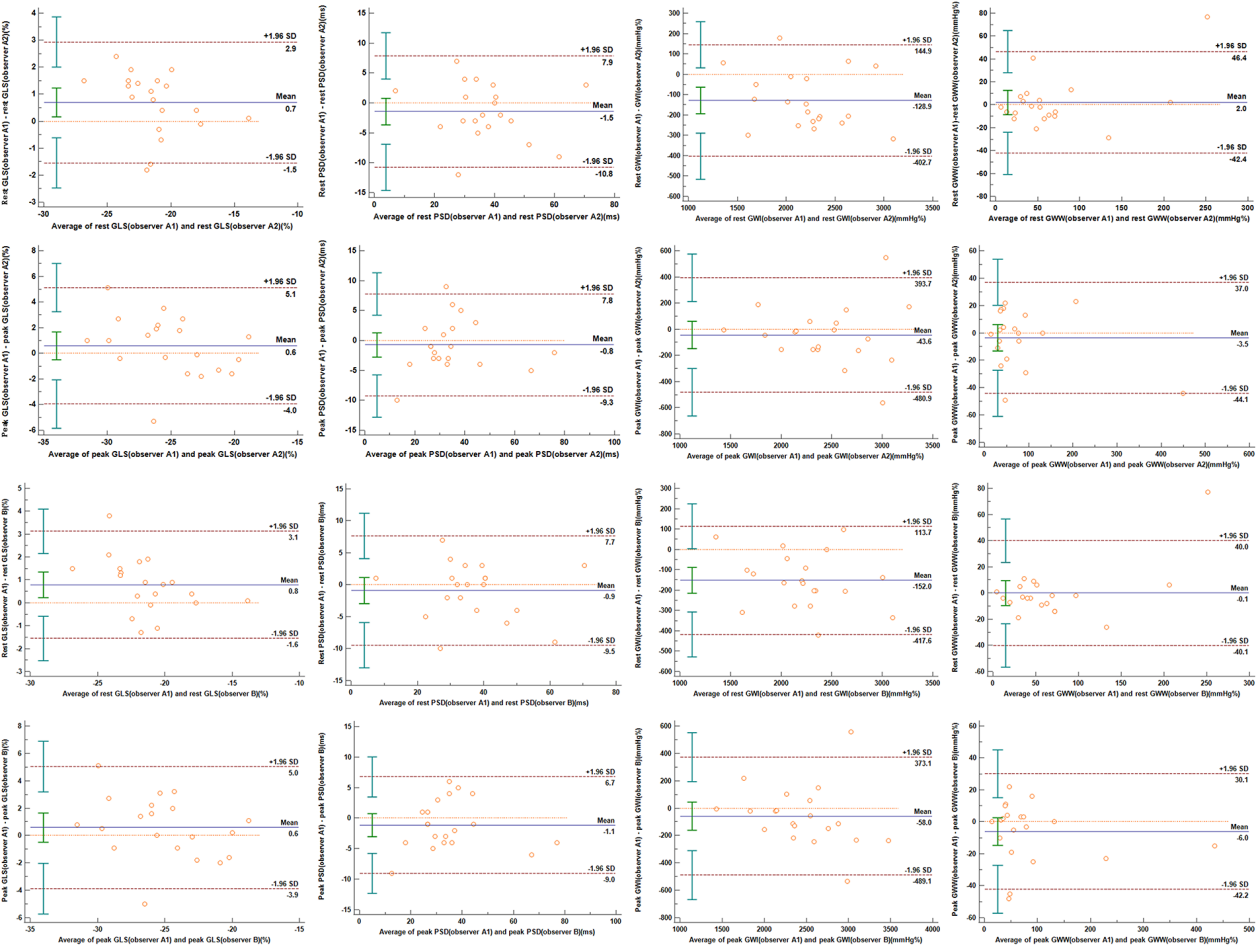
Bland–Altman plots indicating intra-observer and inter-observer variabilities in 2D-STE and MW indices. The upper and lower dotted lines indicate 95% limits of agreement, while the middle dotted line indicates the zero line. The solid line represents the mean difference between the two measurements. (**A**) Intra-observer variability; (**B**) interobserver variability. GLS, global longitudinal strain; PSD, peak strain dispersion; GWI, global work index; GWW, global waste work.

Excellent intra-observer and inter-observer variabilities were observed while measuring the MW parameters (Figure 5). For the intra-observer variability, the interclass correlations coefficients (ICC) of rest GLS, rest PSD, rest GWI, rest GWW, peak GLS, peak PSD, peak GWI and peak GWW were found to be 0.912, 0.941, 0.950, 0.937, 0.907, 0.957, 0.946 and 0.977 respectively. For the inter-observer variability, the ICC of rest GLS, rest PSD, rest GWI, rest GWW, peak GLS, peak PSD, peak GWI and peak GWW were found to be 0.954, 0.974, 0.976, 0.973, 0.956, 0.982, 0.952 and 0.991, respectively.

## 4. Discussion

This study is a first and comprehensive exploration of the effects of CMD on cardiac systolic function, including deformation capacity, myocardial work and cardiac contraction synchronization, in ANOCA patients. The main findings of this study are as follows: (1) Compared to the non-CMD group, patients in the CMD group had impaired contractility and systolic synchronization under stress. (2) Patients in the CMD and non-CMD groups responded differently to adenosine. In the CMD group, the changes in MW under adenosine stress were mainly manifested as an increase in GWW and a decrease in GWE, while, in the non-CMD group, the main changes in MW upon adenosine stress were an increase in GWI, GCW and GWE and a decrease in GWW. (3) GWI and GCW were mainly associated with SBP, DBP, RPP, GLS and coronary flow velocity. While GWW was mainly correlated with PSD, GWE was correlated with PSD and GLS. (4) Multivariate regression analysis showed that ΔPSD and ΔGWW were independently associated with CMD. 2D-STE related indices and MW had good predictive value for CMD, and the combined forecasting metric consisting of ΔPSD and ΔGWW demonstrated the best predictive value for CMD with an AUC of 0.923. Our study is the first to investigate the effect of CMD on MW in ANOCA patients and to investigate the difference in the response to adenosine between CMD and non-CMD ANOCA patients. It also demonstrates that LV mechanics has good predictive value for CMD.

Several studies have reported the presence of CMD in approximately 25%–65% of ANOCA patients, which patients have a worse prognosis than ANOCA patients without CMD (17–20). The presence of CMD can cause demand myocardial ischemia and subendocardial fibrosis, which can impair cardiac function. In the iPOWER study, which focused on female ANOCA patients, Jakob et al. found that although CMD did not cause more severe angina symptoms, it was associated with a higher incidence of adverse cardiovascular events (HR: 1.94), with heart failure and coronary nonobstructive myocardial infarction (MINOCA) being the main factors associated with adverse outcomes in ANOCA patients with combined CMD (21). In a multicenter study of patients with nonischemic heart failure, Clarissa et al. found that patients with HFrEF had lower CFR values than those with HFpEF and that reduced LVEF was an independent correlate of CMD (22). In addition, numerous studies have confirmed the correlation between CMD and heart failure markers (23, 24). All of the above studies confirm that CMD plays a vital role in the development of cardiac insufficiency. However, noninvasive echocardiography has difficulty in identifying systolic dysfunction in ANOCA patients with CMD. In addition, previous studies have focused on the effect of CMD on diastolic function, not systolic function. Therefore, we designed this research to investigate the changes in cardiac systolic function by noninvasive echocardiography with a new method in ANOCA patients with CMD, to explore the predictive index of ANOCA.

### 4.1. Impact of CMD on 2D-STE indices

Most cardiovascular risk factors, such as advanced age, obesity, diabetes, and hyperlipidemia, promote the development of a systemic proinflammatory state and the accumulation of reactive oxygen species in the vascular endothelium, thereby causing an inflammatory response in the coronary microvascular endothelium and the extracellular interstitium of the myocardium, which are considered the pathophysiological basis of CMD. The inflammatory response in the myocardial extracellular interstitium promotes myocardial fibrosis and remodeling of the extracellular matrix, which reduces the elasticity of the heart and causes changes in GLS, an indicator of cardiac deformation (3, 25). In addition, myocardial interstitial fibrosis induces myocardial heterogeneous activation, and slows conduction in fibrotic areas, thereby impairing the synchronization of electromechanical conduction and causing deterioration of indicators of cardiac contraction synchronization such as PSD (7). As our findings demonstrated, the absolute value of GLS was lower in the CMD group than in the non-CMD group after adenosine stress, which is consistent with the findings of Hugo Rodriguez et al. and Tagliamonte et al. (26, 27).The CMD group also had impaired synchronization of cardiac contractions.

However, as adenosine is a vasodilator, it has potential effects on arterial blood pressure, and the load-dependent properties of 2D-STE-related indices dictate that they are not well interpreted under conditions of blood pressure variability.

Therefore, our study also explored the responsiveness of ANOCA patients with and without CMD to adenosine, which is a good complement to previous studies. In terms of hemodynamic indices, the CMD group showed no significant change in blood pressure after adenosine stress and a significant increase in RPP, but the non-CMD group showed a significant decrease in blood pressure. As for 2D-STE related indices, there was no significant change in PSD from before to after adenosine stress in the CMD group, but peak PSD was significantly lower than resting PSD in the non-CMD, indicating that adenosine improved cardiac systolic synchronization in the non-CMD group but not in the CMD group. Recent studies have confirmed that, under similar pathophysiological mechanisms, ANOCA patients with CMD often have combined peripheral vascular endothelial cell dysfunction, resulting in peripheral vasodilator dysfunction similar to that of coronary microvascular dysfunction (28, 29). Our results corroborate this idea. Recent studies have confirmed that many diseases that cause systemic inflammation can lead to CMD (30). In fact, in recent years, some scholars have viewed CMD as a cardiac manifestation of systemic disease. Cardiac fibrosis caused by CMD (31), especially interstitial fibrosis, leads to a reduced sensitivity to altered cardiac synchrony, which is why PSD in the CMD group did not change significantly before and after stress.

### 4.2. Impact of CMD on MW

We further explored the effect of CMD on MW and the factors influencing MW. Since noninvasive MW has proven reliable, its application in ischemic heart disease is valid. For example, Natalie et al. demonstrated that GWI, GCW, and GWE were significantly reduced in patients with obstructive coronary artery disease (32), and Rodolfo et al. demonstrated that in STEMI patients treated with primary PCI, MW in the culprit vessel territory is independently associated with early adverse LV remodeling (33). However, an analysis of the effect of CMD as a type of ischemic heart disease on MW has been lacking relevant studies. Our study showed no significant difference in MW between ANOCA patients with and without CMD at rest. However, after adenosine administration, GWI, GCW, and GWE were lower in the CMD group than in the non-CMD group, while GWW was higher in the CMD group.

MW is an indirect measure of cardiac contractility and myocardial oxygen consumption, and our study confirms that this is mainly reflected in GWI and GCW. While ΔSBP, ΔDBP and ΔRPP were similar in the CMD and non-CMD groups, the difference between peak GWI and peak GCW should be attributed to the difference in contractility at stress between the two groups, which was corroborated by the differences in peak GLS and ΔGLS between the two groups. Peak GWW and ΔGWW showed a positive correlation with peak PSD and ΔPSD, so we considered that higher GWW in the CMD group at stress was associated with cardiac contractile dyssynchrony. Thus, we believe that 2D-STE related indices and MW have potential clinical value in the diagnosis of ANOCA with CMD.

However, MW is not independent of blood pressure. Tsai et al. (11) showed a correlation between MW and blood pressure in their study of untreated hypertensive patients. They found that compared to controls, hypertensive patients had significantly higher GWI and GCW, but lower GWE. In the correlation analysis, Tsai et al. found a significant positive correlation between GWI and GCW with blood pressure, which is identical to our results and further corroborates our conclusions. Our study showed that patients in the non-CMD group had a significant decrease in blood pressure at stress, while GWI and GCW increased significantly, whereas patients in the CMD group with no change in blood pressure had no significant increase in GWI or GCW. This suggests that ANOCA patients with CMD have lost cardiac contractile reserve capacity at stress, a manifestation of subclinical impairment of cardiac systolic function. The changes in MW after adenosine stress in ANOCA patients with CMD in the present study followed the same pattern of changes in MW in patients with positive exercise stress echocardiograms in the study by Andrew et al. (34). Our findings further reveal the essence of CMD as ischemic heart disease, which is also supported by the relationship between CMD and markers of myocardial injury (35, 36). As for GWW and GWE, since their correlation with blood pressure was not found in the correlation analysis, their changes could be considered to be related to changes in the synchrony of cardiac contraction.

### 4.3. Predictors of CMD

PSD is a strain-based quantification of the time synchronization of electromechanical conduction and an important index for the study of cardiac systolic function (7). Abnormal electromechanical synchronization decreases the mechanical efficiency of left ventricular ejection, increases energy loss and ineffective work, and reduces global work and efficiency. Previous studies have confirmed the significant alteration and prognostic value of PSD in various cardiovascular diseases such as hypertrophic cardiomyopathy and aortic stenosis, and its correlation with patients' symptoms (7, 37). Our multivariate regression analysis indicated that ΔPSD and ΔGWW were independently associated with CMD, suggesting that altered cardiac systolic synchrony and increased wasted work under stress may be the main effects of CMD on cardiac systolic function. For predicting CMD, both ΔGWW and ΔPSD showed high predictive value, while the joint predictor consisting of both demonstrated superior predictive power (AUC: 0.923). Although GWW is thought to be associated with cardiac contraction asynchrony, there has been a lack of data from relevant clinical studies to support this hypothesis, but our findings support it.

In summary, our study shows that the changes in cardiac systolic function in ANOCA patients with CMD after adenosine stress are mainly manifested as reduced systolic synchronization and increased useless work, resulting in reduced cardiac contraction efficiency and increased energy depletion, which eventually lead to impaired cardiac function. These findings provide important targets and ideas for the early diagnosis and treatment of ANOCA patients with CMD.

## 5. Limitations

Our results should be interpreted considering some limitations. First, this study is a single-center retrospective study with a small sample size. A large, prospective study should be conducted in the future. Second, our study only explored cardiac systolic function, ignoring alterations in diastolic function. In fact, previous studies have confirmed the effect of CMD on cardiac diastolic function; for example, a study with diabetic patients confirmed that *E*/*e*′ was higher in diabetic patients with CMD (38), and the PROMIS-HFpEF trail confirmed that CMD was associated with reduced left atrial strain (23). Recent study found that left atrial reservoir strain was lower in diabetic patients, confirming the presence of a subclinical diastolic dysfunction associated to the microcirculatory impairment (30). Third, adenosine mainly reflects non-endothelial-cell dependent diastolic dysfunction, but the evaluation of endothelial cell-dependent diastolic function and microvascular spasm was neglected. Fourth, long-term follow-up of patients was not performed. Fifth, regional MW should be calculated and compared with global MW. Last, we considered impaired synchronization of cardiac contraction to result from CMD-induced cardiac fibrosis, but related theories have been put forth by other scholars, and cardiac MRI or serum markers were not measured in this cohort to determine the extent of fibrosis.

## 6. Conclusion

In the present study, we found that CMD caused deterioration of cardiac systolic function in ANOCA patients under adenosine stress and that increased cardiac contraction asynchrony and wasted work may be the main changes caused by CMD.

## Data Availability

The original contributions presented in the study are included in the article/supplementary material, further inquiries can be directed to the corresponding author/s.

## Supplementary material

Correlation of hemodynamic, 2D-STE, and conventional echocardiographic parameters with changes of MW parameters
