# Supplementary material for: The value of myocardial work in the estimation of left ventricular systolic function in patients with coronary microvascular disease: A study based on adenosine stress echocardiography
Source: Front Cardiovasc Med. 2023 Apr 11;10:1119785. doi: 10.3389/fcvm.2023.1119785 (PMC10126338; doi:10.3389/fcvm.2023.1119785)
Supplement: Correlation of hemodynamic, 2D-STE, and conventional echocardiographic parameters with changes of MW parameters [file Table1.docx]

**Supplementary Table 1 Correlation of hemodynamic, 2D-STE, and conventional echocardiographic parameters with changes of MW parameters**

|  | ∆GWI | | ∆GCW | | ∆GWW | | ∆GWE | |
| --- | --- | --- | --- | --- | --- | --- | --- | --- |
|  | r | p | r | p | r | p | r | p |
| ∆SBP | 0.573 | ＜0.001 | 0.478 | ＜0.001 | -0.196 | 0.085 | 0.282 | 0.012 |
| ∆DBP | 0.466 | ＜0.001 | 0.347 | 0.002 | -0.213 | 0.061 | 0.200 | 0.080 |
| ∆HR | -0.288 | 0.011 | -0.261 | 0.021 | 0.060 | 0.640 | -0.085 | 0.461 |
| ∆RPP | 0.208 | 0.049 | 0.106 | 0.354 | -0.050 | 0.665 | 0.101 | 0.377 |
| ∆GLS | -0.645 | ＜0.001 | -0.664 | ＜0.001 | 0.083 | 0.469 | -0.285 | 0.011 |
| ∆PSD | -0.119 | 0.263 | -0.142 | 0.214 | 0.376 | 0.001 | -0.429 | ＜0.001 |
| ∆LVEF | 0.139 | 0.225 | 0.216 | 0.058 | 0.060 | 0.602 | 0.045 | 0.698 |
| ∆LVEDVI | -0.043 | 0.706 | -0.071 | 0.538 | 0.037 | 0.746 | -0.090 | 0.432 |
| ∆LVESVI | -0.168 | 0.141 | -0.245 | 0.031 | -0.058 | 0.616 | -0.064 | 0.580 |
| ∆ Coronary flow velocity | 0.052 | 0.649 | 0.021 | 0.854 | -0.111 | 0.332 | 0.122 | 0.288 |

SBP, systolic blood pressure; DBP, diastolic blood pressure; HR, heart rate; RPP, product of heart rate and blood pressure; GLS, global longitudinal strain; PSD, peak strain dispersion; LVEF, left ventricular ejection fraction; LVEDVI, left ventricular end-diastolic volume index; LVESVI, left ventricular end-systolic volume
